# Iatrogenic complications following shunting the subarachnoid space in infancy

**DOI:** 10.1186/1743-8454-7-S1-S13

**Published:** 2010-12-15

**Authors:** Douglas Cochrane, Ryan Janicki, Keith Aronyk, Jeff Pugh, Paul Steinbok, Ash Singhal

**Affiliations:** 1 Department of Surgery, Division of Neurosurgery, University of British Columbia, Vancouver, BC, Canada; 2 Department of Surgery, University of Alberta, Edmonton, AB, Canada

## Background

Of those patients who require extracranial shunting to manage CSF circulation disorders, some of the most challenging are those whose shunts have been placed in the subarachnoid space in infancy or childhood. While clinically well in childhood, with few if any revisions, a symptom complex may develop in mid to late childhood that is characterized by chronic, often non-specific headaches and intermittent intracranial hypertension associated with abducens palsy and/or papilloedema. Imaging shows some combination of decompressed CSF spaces, acquired hindbrain herniation or posterior fossa crowding, diploic hypertrophy, narrow spinal canal and meningeal thickening. ICP monitoring does not always provide clarification of the clinical ICP correlation.

## Materials and methods

This paper describes five patients with acquired hindbrain herniation, three following subarachnoid shunting for cerebral arachnoid cyst. Various treatments, including shunt revision, shunt valve adjustments and hindbrain hernia decompression, were performed without enduring effectiveness.

## Results

Based on this experience, the authors propose that the evolution of craniocerebral/cerebellar distortion due to loss of the distending forces of the subarachnoid space in early childhood results in central chronic transtentorial shifts that impact venous drainage at the level of the galenic and petrosal systems. There is a failure to shift cerebral venous return from the dural sinus system to the basilar and condylar plexuses with postural changes with or without dural sinus compression. Chronic venous insufficiency results in swelling of posterior fossa structures. Hindbrain herniation and/or crowding is the result.

## Conclusions

Prevention of this complication can be achieved by avoiding subarachnoid shunts in this age group and utilizing other options to address arachnoid cysts that are deemed to need treatment. In those patients who are symptomatic, reconstruction of the subarachnoid space by extensive supratentorial cranial expansion may be effective based on relief of transtentorial brain shifts and consequent venous congestion.

